# A collection of Aquamaps native layers in NetCDF format

**DOI:** 10.1016/j.dib.2018.01.026

**Published:** 2018-01-31

**Authors:** Paolo Scarponi, Gianpaolo Coro, Pasquale Pagano

**Affiliations:** a Researcher at ISTI-CNR, via Giuseppe Moruzzi 1, Pisa, PI, Italy; b Senior Researcher at ISTI-CNR, via Giuseppe Moruzzi 1, Pisa, PI, Italy

## Abstract

Many research communities working in biology and related fields are deeply interested in having a wide collection of environmental and species distribution data. Obviously, for these communities to be able to carry out their studies in a fast and efficient manner, these data need to be well organized, meticulously described and possibly represented in a standard format that allows for a direct usage. In fact, being the final goal of these communities to extract information from the data and applying some kind of data processing workflows, cutting down the data preparation and preprocessing time is key. This is the main reason that triggered the activity presented in this paper that aimed at converting the whole collection of 10,385 species distribution models published by the Aquamaps consortium (Aquamaps Native Distributions) into NetCDF files, creating a re-usable, portable and self-describing collection of native habitat datasets.

**Specifications Table**

**Table:** 

Subject area	*Marine biology*
More specific subject area	*Marine species distribution*
Type of data	*Global bi-dimensional maps*
How data was acquired	*Converted from a previously available collection*
Data format	*NetCDF embedding metadata*
Experimental factors	*A collection of 10.385 marine species habitat distribution maps have been downloaded in a polygon CSV format and converted in a standard latitude-longitude CSV format*
Experimental features	*Experimental features & The newly generated latitude-longitude CSV datasets have been converted into metadata-enhanced NetCDF files and published onto an online repositories*
Data source location	*Every dataset in the collection is a latitude-longitude probability map, the geo-reference for each probability value is inside the datasets themselves*
Data accessibility	*The collection of NetCDF files is published onto THREDDS at the following address:*http://thredds.d4science.org/thredds/catalog/public/netcdf/AquamapsNative/catalog.html*[be careful to copy the whole link, including the "catalog.html" part, otherwise it does not work].*
	*As an alternative way to access the data follow this link:*http://thredds.d4science.org/thredds/catalog.html*[then navigate to "public_files/AquamapsNative".]*
	*The dataset collection has been also made accessible through the D4Science Infrastructure's Data Catalogue at:*https://ckan-bb2.d4science.org/organization/d4sciencelabs
	*The metadata have been registered into GeoNetwork at:*http://geonetwork.d4science.org/geonetwork/srv/en/main.home

**Value of the data**

This datasets collection is very useful to many research communities for the following reasons, mainly related to the NetCDF format nature:

• Every piece of data is a standalone machine-independent file, and thus extremely easy to reuse and share.
• There are plenty of tools to visualize and process NetCDF file and for this reason the data are extremely easy to use and do not require any preprocessing.
• The NetCDF format allows a-posteriori variables addition, making each dataset a potentially expandable entity that can be updated and enhanced in time.

## 1 Data

This data collection consists of 10,835 datasets, each one representing a habitat distribution map for a specific marine species. The resolution is equal to 0.5° on both latitude and longitude, while to each point in the globe has been associated a value between 0 and 1, corresponding to the probability of such species to be found in that point. Those maps have been generated using a specific model that we describe in the next section of this document and that process environmental variables to predict the habitat suitability for a given species.

## 2 Experimental design, materials and methods

We first itemize all the standard and tools we used to produce this collection of datasets and then we describe the procedure we put in place in a detailed manner.

### 2.1 Tools and standards

• *Aquamaps*: Aquamaps is a set if models that generate large-scale predictions of species natural suitability based on environmental data [6]. These models are capable of exploiting only the information about presence points for the species (no absence points can be included) and incorporate scientific expert knowledge to account for known biases and limitations of marine species occurrence records [8]. Aquamaps includes a model (named Native) to estimate the actual distribution of a species and another model (named Suitable) to estimate 'potential' habitat suitability in locations where the species has never been observed. Aquamaps produces polygonal maps, meaning that the space is divided into polygons to which a single value is associated for a certain parameter. In particular, the polygons are usually *1.0°* × *1.0°* squares, thus the spatial resolution is equal to 0.5°. One of the main reasons that lead us to use these data is the reliability of the Aquamaps modeling approach with respect to other approaches (especially when expert knowledge is included in the model) and thus, the quality of the produced datasets. Moreover, our research group has been part of many European projects involving the Food and Agriculture Organization (FAO) and other organizations that endorse this model, thus we had a large number of these Aquamaps-generated datasets easily available for our purposes.
• *NetCDF*: The Network Common Data Format (NetCDF) is a self-describing, machine-independent data format that is meant to represent and store array-oriented data [9], [10]. The NetCDF format, on top of being self-describing and designed to represent n-dimensional data with *n* ≥ *2*, is also widely used by many communities and research institutions as a standard. Further, many tools and libraries written in a large variety of programming languages are available to visualize, manipulate, and process this format. Moreover, additional information about the data can be included in the file itself as attributes, creating more complex objects that do not need any external reference to be fully understandable, and thus reusable and portable. For all these reasons we decided to use this data format to represent all the Aquamaps Native Distributions.
• *THREDDS*: The Thematic Real-Time Environmental Distributed Data Service (THREDDS) is a web-service meant to provide students, educators and researchers with distributed access to a large collection of real-time and archived datasets from a variety of environmental data sources [5]. We published our data in a custom-made repository in order to make them easily accessible to the general public. One of the main reasons why we chose this tool is its versatility in terms of access protocols, among which: OPeNDAP DAP2, NetCDF Subset Service, OGC Web Coverage Service (WCS) and OGC Web Map Service (WMS). Indeed, having many way of accessing the data make them more usable and shareable.
• *GeoNetwork*: GeoNetwork is a standardized and decentralized spatial information management service that allows users to publish and browse metadata items associated to any kind of geo-referenced dataset [11]. We decided to publish all the products' metadata in this catalog to provide an easy and widely used access point (in addition to THREDDS and the its supported protocols) as well as to enrich the data with some additional metadata.
• *D4Science Infrastructure*: D4Science is a Hybrid Data e-Infrastructure meant to provide scientists and researchers in many different communities with collaborative 'workplaces' called Virtual Research Environments (VREs, see [1]). These VREs are equipped with a large number of facilities that implement the following functionalities:
  ● Distributed storage
  ● Parallel and distributed data processing
  ● Data publication, visualization, browsing, manipulation and access
  ● Security, authorization and accounting
  ● Social networking

In this section we will briefly describe only the two services we exploit the capabilities of during our activity, namely the DataMiner and the D4Science Data Catalogue.

• *DataMiner*: DataMiner is an open source computational system which is able to interoperate with the other services of the D4Science Research e-Infrastructure [2]. It uses the Web Processing Service (WPS, see [4]) standard to publish/describe the hosted processes and produces a provenance XML file for each experiment in the Prov-O ontological format [7]. DataMiner also implements a Map-Reduce [3] approach for Big Data processing and saves inputs, outputs, and provenance information onto a collaborative experimentation space that supports the sharing of this information between different users.
• *D4Science Data Catalogue*: The D4Science Data Catalogue contains links to resources produced by the e-Infrastructure users in a number of domains, ranging from computational biology to cultural heritage and scientific training. The indexed data include species distribution maps, environmental data, and area regulation zones. All the products are the result of joint collaborations between several research institutes that have used D4Science and are accompanied by rich descriptions capturing general attributes such as the title, the creators, the usage policies, the licenses and so on. More information about the Data Catalogue can be found at https://services.d4science.org/catalogue.

### 2.2 Method description

The main idea behind this work was to create a huge repository of environmental and species distribution data in a re-usable and standard format. The goal was to simplify the usability of these data pushing to zero the pre-processing effort, embedding proper descriptions into the datasets themselves to create standalone objects, thus allowing different communities to easily produce new information out of them as well as sharing them. Further, we aimed at formalizing the approach while defining a reproducible workflow to convert existing datasets into those standalone entities that could enhance the longevity and the re-usability of the data. Our approach to the specific data conversion task of producing NetCDF representations for the Aquamaps distributions can be easily customized to process other environmental data and species distribution layers collections, and it is made up of the following steps:

1. Programmatically download from GeoServer all the Aquamaps Native Maps in CSV format via WFS requests
2. Convert every CSV polygon map into a CSV latitude-longitude one, using the center of mass for every square
3. Convert the latitude-longitude CSV maps into a NetCDF files using an automatic procedure. In our case we used a DataMiner process called “CSV TO NETCDF CONVERTER XY” available at: https://services.d4science.org/group/rprototypinglab/data-miner?OperatorId=org.gcube.dataanalysis.wps.statisticalmanager.synchserver.mappedclasses.transducerers.CSV_TO_NETCDF_CONVERTER_XY
4. Programmatically upload the new NetCDF files onto THREDDS and register metadata on GeoNetwork. These two task have been accomplished by means of another DataMiner process called “RASTER DATA PUBLISHER”, available at https://services.d4science.org/group/rprototypinglab/data-miner?OperatorId=org.gcube.dataanalysis.wps.statisticalmanager.synchserver.mappedclasses.transducerers.RASTER_DATA_PUBLISHER, that applies them sequentially

We validated the conversion quality by comparing the new layers with the old layers using an automatic procedure involving a point to point comparison between the original maps and the new maps, using the Euclidian Norm of the difference between the maps to measure the disagreement (a perfect zero disagreement confirmed the correct conversion of the original maps).
